# Pecan Intake Improves Lipoprotein Particle Concentrations Compared with Usual Intake in Adults at Increased Risk of Cardiometabolic Diseases: A Randomized Controlled Trial

**DOI:** 10.1016/j.tjnut.2025.03.014

**Published:** 2025-03-18

**Authors:** Tricia L Hart, Penny M Kris-Etherton, Kristina S Petersen

**Affiliations:** Department of Nutritional Sciences, Pennsylvania State University, University Park, PA, United States

**Keywords:** pecans, nuts, apolipoprotein B, lipoprotein subfractions, LP-IR, insulin resistance

## Abstract

**Background:**

Pecan consumption consistently improves lipoproteins, but less research has investigated the effect of pecans on lipoprotein subfractions.

**Objectives:**

The aim was to investigate the effect of substitution of usual snack foods with 57 g/d of pecans on lipoprotein particle subfractions and apolipoproteins compared with continuing usual intake after 12 wk. Exploratory analyses evaluated effects on early markers of insulin resistance including the Lipoprotein Insulin Resistance Index (LP-IR), Diabetes Risk Index, and GlycA.

**Methods:**

A 12-wk, randomized, 2-armed parallel trial in adults at risk of cardiometabolic disease was conducted. Participants were instructed to either consume 57 g/d of pecans in place of usual snacks or to continue their usual intake. Plasma samples collected at baseline and 12 wk were analyzed for lipoproteins, apolipoproteins, and GlycA by proton nuclear magnetic resonance spectroscopy. Between-group differences in the change from baseline were evaluated with linear regression.

**Results:**

In total, 138 participants were randomly assigned (*n* = 69 per group) and 130 participants (pecan group *n* = 62; usual diet group *n* = 68) completed the trial. The pecan group had a greater reduction from baseline in the concentrations of apolipoprotein B (apoB) [–4.38 mg/dL; 95% confidence interval (CI): –8.02, –0.73], total low-density lipoprotein particles (–75.3 nmol/L; 95% CI: –144, –6.93), total triglyceride-rich lipoprotein particles (TRL-P) (–20.4 nmol/L; 95% CI: –33.8, –7.03), large (–1.47 nmol/L; 95% CI: –2.69, –0.26) and small (–11.3 nmol/L; 95% CI: –22.4, –0.27) TRL-P and the LP-IR (–4.42 points; 95% CI: –8.14, –0.69), and greater increases from baseline in the concentration of large high-density lipoprotein particles (0.35 μmol/L; 95% CI: 0.07, 0.63) compared with the usual diet group.

**Conclusions:**

Incorporating 57 g/d of pecans into the diet in place of usual snacks for 12 wk improved apoB, atherogenic lipoprotein subfractions, and the LP-IR in adults at risk of cardiometabolic diseases.

This trial was registered at clinicaltrials.gov as NCT05071807.

## Introduction

Consistent clinical trial evidence shows that pecan intake improves LDL cholesterol and non-HDL cholesterol concentrations to a clinically relevant degree [[1], [2], [3], [4]]. LDL cholesterol and non-HDL cholesterol are clinically measured surrogates for the number of circulating apolipoprotein B (apoB)-containing lipoprotein particles [5]. All apoB-containing lipoproteins, including LDL particles and triglyceride-rich lipoprotein particles (TRL-P) (i.e., VLDL particles and chylomicrons), are atherogenic and increase the risk of atherosclerotic cardiovascular disease (CVD) [5]. Each apoB-containing lipoprotein particle contains 1 apoB molecule; therefore, apoB concentration is highly correlated with the concentration of LDL cholesterol and non-HDL cholesterol [5]. However, at the individual level, discordance is often observed between apoB and LDL cholesterol/non-HDL cholesterol because of variation in the mass of cholesterol within the apoB-containing lipoproteins [5]. In addition, discordance is common in individuals with metabolic syndrome (MetS) [6]. MetS is a constellation of interrelated risk factors for CVD and type 2 diabetes, which is characterized by insulin resistance, inflammation, and atherogenic dyslipidemia [7]. The effect of pecan intake on apoB-containing lipoproteins has not been well investigated in adults with features of MetS.

Clinical trial evidence shows that nut consumption improves peripheral insulin resistance assessed by the HOMA-IR [8]. Insulin resistance precedes dysglycemia and prediabetes onset and results in progressive loss of beta-cell function [9]. Lipoprotein particle sizes and concentrations are altered in insulin resistance [9]. In recognition, the Lipoprotein Insulin Resistance Index (LP-IR) was created to assess insulin resistance based on lipoprotein abnormalities characteristic of insulin resistance (i.e., higher concentrations of large TRL-P and small LDL particles, lower concentrations of large HDL particles, smaller size LDL and HDL particles, and larger size TRL-P) [[10], [11], [12]]. Findings from several prospective cohort studies show that the LP-IR is positively associated with type 2 diabetes risk [10,[13], [14], [15]]. Subsequently, the Diabetes Risk Index (DRI) was developed to further improve type 2 diabetes risk prediction. The DRI is calculated based on the LP-IR and concentrations of branched-chain amino acids valine and leucine [16]. The DRI predicts the future risk of type 2 diabetes and improves type 2 diabetes risk classification compared with the LP-IR [16]. Finally, GlycA is a novel biomarker of systemic inflammation linked to abnormal insulin metabolism and predicts incident type 2 diabetes independently of traditional risk factors [17,18]. Previously, a randomized controlled trial showed pecan intake improved HOMA-IR and beta-cell function (HOMA-β) [19]. To the authors’ knowledge, the effect of pecan intake on lipoprotein-related early markers of insulin resistance has not been investigated.

We recently conducted a 12-wk clinical trial examining the effect of consuming 57 g/d of pecans in place of usual snacks compared with continuing usual intake on vascular health and risk factors for cardiometabolic diseases [20]. In this trial, pecan intake reduced total cholesterol [–8.1 mg/dL; 95% confidence interval (CI): –14.5, −1.7], LDL cholesterol (–7.2 mg/dL; 95% CI: –12.3, –2.1) and non-HDL cholesterol (–9.5 mg/dL; 95% CI: –15.3, –3.7) compared with the usual diet group [20]. Therefore, the aim of this secondary analysis was to investigate how instructions to substitute usual snack foods with 57 g/d of pecans affect the concentration of lipoprotein particle subfractions, lipoprotein particle size, and apolipoproteins compared with continuing usual intake after 12 wk in adults at risk of cardiometabolic diseases. Exploratory analyses were conducted to examine the effect of the intervention on early markers of insulin resistance including the LP-IR, DRI, and GlycA. We hypothesized that consuming pecans in place of usual snacks would result in a shift in the concentration of apolipoproteins and lipoprotein subfractions that is reflective of lower cardiovascular disease risk and less insulin resistance compared with continuing usual intake.

## Methods

### Study design

A 12-wk, 2-arm parallel randomized controlled trial was conducted at the Pennsylvania State University. The trial methods and results of the primary outcome have been described elsewhere [20]. Participants with 1 or more features of MetS were randomly assigned via a computer-generated randomization scheme (randomization.com) in a 1:1 ratio to 1 of 2 groups: *1*) provided 57 g/d of pecans to consume in place of usual snacks; and *2*) continue usual intake. The primary outcome of this trial was flow-mediated dilation and lipoprotein subfractions were prespecified secondary outcomes. The insulin resistance markers are exploratory outcomes. EDTA plasma samples were analyzed for lipoprotein particle subfractions, apolipoproteins, GlycA, and branched-chain amino acids. The institutional review board at the Pennsylvania State University approved the protocol and all participants provided informed consent. This trial is registered at clinicaltrials.gov (NCT05071807).

### Participants

Inclusion and exclusion criteria have been described previously [20]. Briefly, generally, healthy males and females between the ages of 25 and 70 y with a BMI of 25–40 kg/m^2^ free of chronic diseases were eligible. Additionally, participants were required to have ≥1 component of MetS (i.e., waist circumference ≥94 cm males or ≥80 cm females; triglycerides ≥150 mg/dL; HDL cholesterol ≤40 mg/dL male or ≤50 mg/dL females; systolic blood pressure ≥130 mmHg or diastolic blood pressure ≥85 mmHg; and fasting plasma glucose ≥100 mg/dL) [21] to be enrolled. Participants were not taking medications or supplements known to influence study outcomes.

### Outcome assessment

Participants attended the Penn State Clinical Research Center at baseline and after ∼12 wk. To standardize measures, participants were instructed to abstain from alcohol for 48 h, abstain from food, caffeinated beverages, exercise for 12 h, and avoid over-the-counter medications for 24 h. Fasted blood samples were collected by registered nurses in EDTA vacutainers that were centrifuged at 1590 × *g* (±90) for 15 min at room temperature immediately after collection. Plasma samples were aliquoted (0.5 mL) into cryovials and stored at –80° Celsius until analysis. The concentration of lipoprotein subfractions [LDL particles (total, large, medium, and small), TRL-P (total, very large, large, medium, small, and very small), HDL particles (total, large, medium, and small), HDL particle subspecies (H1–H7)], apolipoprotein A, apoB, and GlycA was measured via proton nuclear magnetic resonance spectroscopy by a commercial laboratory using the LipoProfile-4 algorithm [[22], [23], [24]]. Average particle size for LDL particles, TRL-P, and HDL particles was measured by summing the diameter of each subclass multiplied by the relative mass percent [[22], [23], [24]]. The LP-IR was calculated according to a previously published algorithm [10]. The DRI was calculated using the following equation: DRI = 0.0167 (LP-IR) + 1.907 [ln (valine + 2 ∗ leucine)] [16].

### Statistical analysis

All statistical analyses were performed in the statistical analysis system (SAS) (version 9.4; SAS Institute Inc.). The normality of the residuals was assessed by normal probability (Q–Q) plots. Nonnormally distributed variables were log-transformed for analysis, which improved the distribution. In the primary analysis, the between-group difference in the change from baseline for each lipoprotein outcome was evaluated by linear regression (PROC GLM) with adjustment for prespecified covariates: baseline value, age (years), sex (male or female), and BMI (kg/m^2^). In a secondary analysis, the between-group difference at 12 wk was examined using linear mixed models (PROC MIXED) with randomization as a fixed effect and visit as a repeated effect. These models were also adjusted for prespecified covariates: baseline value, age, sex, and BMI. When a main effect of randomization by visit was detected, post hoc pairwise comparisons were conducted, and the Tukey–Kramer method was used to adjust for multiple comparisons. When the randomization by sex interaction was not significant, the interaction term was removed from the final model. Data are presented as least squares mean ± SEM or least squares mean (95% CI) unless otherwise stated. Statistical significance was set at *P* < 0.05.

## Results

### Participants

A total of 138 participants (mean ± SD; 46 ± 13 y; 29.8 ± 3.7 kg/m^2^) were enrolled and 69 adults were randomly assigned into each group. Endpoint testing was completed by 130 participants (pecan group *n* = 62; usual diet group *n* = 68). Reasons for noncompletion included relocation (*n* = 1), unrelated medical issues (*n* = 3), dislike of study food/portion size (*n* = 3), and time conflicts (*n* = 1). Participant characteristics are presented in Table 1. Compliance was high (i.e., >90%) in both study groups and the weight change from baseline was not different between groups after 12 wks (pecan: 0.7 kg; 95% CI: 0.2, 1.3 compared with usual diet: 0.1 kg; 95% CI: –0.4, 0.6; *P* = 0.07). Details on self-reported dietary intake and snack consumption were previously reported [20]. Briefly, baseline meal and snack intake were similar between groups [20]. In addition, no differences in energy or macronutrient intake between the groups were observed at baseline [20]. At 12 wks, the pecan group consumed a greater percentage of energy intake from fat (7.2%; 95% CI: 4.4%, 10.0%), monounsaturated fat (5.1%; 95% CI: 3.8%, 6.4%), and polyunsaturated fat (2.5%; 95% CI: 1.3%, 3.7%), and had higher fiber intake (3.3 g; 95% CI: 0.7, 5.9), than the usual diet group. The pecan group consumed a lower percentage of energy from carbohydrates (–5.8%; 95% CI: –8.7%, –2.9%) than the usual diet group at 12 wks.

**TABLE 1:** Baseline characteristics of participants overall and by randomization^1^.

Characteristic	Pecan (*n* = 69)	Usual diet (*n* = 69)	Total (*n* = 138)
***n* (% female)**	69 (65)	69 (52)	138 (59)
**Age (y)**	46 ± 12	46 ± 14	46 ± 13
**BMI (kg/m^2^)**	29.7 ± 3.7	29.8 ± 3.9	29.8 ± 3.7
**MetS criteria**			
**Waist circumference (cm)**	97.2 ± 8.8	98.8 ± 10.8	98.0 ± 9.9
**Male**	98.2 ± 8.5	101.7 ± 10.7	100.2 ± 10.8
**Female**	96.7 ± 9.1	96.2 ± 10.4	96.5 ± 9.6
**SBP (mmHg)**	123 ± 15	125 ± 11	124 ± 13
**DBP (mmHg)**	78 ± 10	79 ± 8	79 ± 9
**HDL cholesterol (mg/dL)**	54 ± 13.7	52 ± 13.1	53 ± 32.4
**Male**	46.6 ± 9.7	46.6 ± 8.1	46.6 ± 8.75
**Female**	57.6 ± 14.0	56.7 ± 15.0	57.2 ± 14.3
**FPG (mg/dL)**	97 ± 9.9	97 ± 9.4	97 ± 9.6
**Triglycerides (mg/dL)**	109 ± 67.4	120 ± 54.9	114 ± 61.6
**Number of MetS criteria**			
**0, *n* (%)**^2^	5 (3.6)	1 (0.7)	6 (4.3)
**1, *n* (%)**	23 (17)	23 (17)	46 (33)
**2, *n* (%)**	19 (14)	29 (21)	48 (35)
**≥3, *n* (%)**	22 (16)	16 (12)	38 (28)

Abbreviations: DBP, diastolic blood pressure; FPG, fasting plasma glucose; MetS, metabolic syndrome; SBP, systolic blood pressure.

1 Data are mean ± SD.

2 Had at ≥1 MetS criterion at screening, but not at baseline.

### Lipoprotein subfractions

The pecan group had a greater reduction from baseline in the concentrations of apoB (–4.38 mg/dL; 95% CI: –8.02, –0.73), total LDL particles (–75.3 nmol/L; 95% CI: –144, –6.93), total TRL-P (–20.4 nmol/L; 95% CI: –33.8, –7.03), large TRL-P (–1.47 nmol/L; 95% CI: –2.69, –0.26), and small TRL-P (–11.3 nmol/L; 95% CI: –22.4, –0.27) compared with the usual diet group (Table 2). Greater increases from baseline in the concentration of large HDL particles (0.35 μmol/L; 95% CI: 0.07, 0.63) and HDL particle subspecies, H5 (0.40 μmol/L; 95% CI: 0.10, 0.62) were observed in the pecan group compared with the usual diet group (Table 2).

**TABLE 2:** Model-based estimates of the between-group differences in lipoproteins and apolipoproteins in the change from baseline in adults at risk of cardiometabolic diseases.

	Pecan		Usual diet		Effect^2^	*P* value^3^
	*n*	Change (95% CI)	*n*	Change (95% CI)		
**Apolipoproteins(mg/dL)**						
**ApoB**	62	–3.45 (–6.10, –0.80)	68	0.93 (–1.56, 3.43)	–4.38 (–8.02, –0.73)	0.02
**ApoA1**	62	3.51 (0.42, 6.60)	68	2.75 (–0.17, 5.66)	0.76 (–3.48, 5.00)	0.72
**LDL particles (nmol/L)**						
**Total**	62	–62.4 (–112, –12.7)	68	12.8 (–34.0, 59.6)	–75.3 (–144, –6.93)	0.03
**Large**	62	–32.5 (–62.5, –2.44)	68	–4.56 (–32.9, 23.8)	–27.9 (–69.3, 13.4)	0.18
**Medium**	62	–35.5 (–88.5, 17.5)	68	–10.0 (–59.8, 39.9)	–25.5 (–98.6, 47.5)	0.49
**Small**	62	19.2 (–48.9, 87.3)	68	17.3 (–46.9, 81.5)	1.86 (–91.8, 95.5)	0.97
**Triglyceride-rich lipoproteinparticles(nmol/L)**						
**Total**	62	–12.0 (–21.8, –2.28)	68	8.40 (–0.80, 17.6)	–20.4 (–33.8, –7.03)	0.003
**Very large**	62	0.05 (–0.06, 0.16)	68	0.01 (–0.09, 0.11)	0.04 (–0.11, 0.19)	0.61
**Large**	62	–0.31 (–1.19, 0.58)	68	1.17 (0.33, 2.00)	–1.47 (–2.69, –0.26)	0.02
**Medium**	62	1.01 (–2.02, 4.05)	68	4.34 (1.48, 7.19)	–3.32 (–7.49, 0.84)	0.12
**Small**	62	–9.84 (–17.9, –1.81)	68	1.48 (–6.10, 9.07)	–11.3 (–22.4, –0.27)	0.04
**Very small**	62	–1.12 (–11.1, 8.84)	68	1.55 (–7.69, 10.8)	–2.66 (–16.3, 11.0)	0.70
**HDL particles(μmol/L)**						
**Total**	62	0.41 (–0.07, 0.88)	68	0.31 (–0.14, 0.76)	0.10 (–0.56, 0.75)	0.77
**Large**	62	0.31 (0.10, 0.51)	68	–0.04 (–0.24, 0.15)	0.35 (0.07, 0.63)	0.02
**Medium**	62	–0.09 (–0.47, 0.28)	68	0.23 (–0.12, 0.59)	–0.33 (–0.85, 0.19)	0.22
**Small**	62	0.19 (–0.38, 0.75)	68	0.16 (–0.37, 0.69)	0.03 (–0.75, 0.80)	0.94
**HDL particle subspecies (μmol/L)**						
**H1**	62	0.21 (–0.14, 0.56)	68	–0.26 (–0.59, 0.07)	0.48 (–0.00, 0.96)	0.05
**H2**	62	–0.03 (–0.56, 0.49)	68	0.40 (–0.09, 0.90)	–0.44 (–1.16, 0.29)	0.24
**H3**	62	0.03 (–0.33, 0.40)	68	0.04 (–0.30, 0.38)	–0.01 (–0.51, 0.50)	0.97
**H4**	62	–0.10 (–0.36, 0.17)	68	0.23 (–0.02, 0.47)	–0.33 (–0.69, 0.04)	0.08
**H5**	62	0.25 (0.06, 0.44)	68	–0.11 (–0.29, 0.07)	0.40 (0.10, 0.62)	0.006
**H6**	62	0.02 (–0.11, 0.15)	68	–0.01 (–0.13, 0.12)	0.03 (–0.15, 0.21)	0.73
**H7**	62	0.05 (–0.02, 0.11)	68	0.04 (–0.02, 0.10)	0.01 (–0.08, 0.09)	0.89
**Average particle size (nm)**	62		68			
**LDL**	62	–0.07 (–0.14, –0.00)	68	–0.03 (–0.09, 0.04)	–0.04 (–0.14, 0.05)	0.37
**Triglyceride-rich lipoprotein**	62	0.80 (–0.78, 2.38)	68	1.65 (0.14, 3.16)	–0.85 (–3.04, 1.34)	0.44
**HDL**	62	0.04 (–0.00, 0.08)	68	0.02 (–0.02, 0.05)	0.02 (–0.03, 0.08)	0.43

Abbreviations: ApoB, apolipoprotein B; CI, confidence interval.

^1^Statistical analyses were performed with SAS version 9.4 (SAS Institute). PROC GLM was used to determine between-group differences with adjustment for baseline value, age, sex, and BMI. Data presented as least squares mean difference (95% CI).

2 Between group mean difference in the change from baseline (95% CI).

3 Main effect of randomization.

At 12 wks, the concentrations of apoB (–4.4 mg/dL; 95% CI: –7.8, –1.0) and total TRL-P (–19.5 nmol/L; 95% CI: –31.9, –7.1) were lower in the pecan group than in the usual diet group (Supplemental Table 1). The concentration of H4 was lower (–0.4 μmol/L; 95% CI: –0.7, 0.0) and H5 was higher (0.3 μmol/L; 95% CI: 0.1, 0.6) in the pecan group after 12 wks than in the usual diet group. For H4, a significant randomization by sex interaction was observed. Post hoc pairwise testing showed that females in the pecan group had lower H4 (–0.4 μmol/L; 95% CI: –0.7, –0.1) than females in the usual diet group, and males in the usual diet group had lower H4 than females in the usual diet group (–0.4 μmol/L; 95% CI: –0.8, –0.1). There were no significant between-group differences at 12 wks for total LDL particles or large TRL-P, which was observed in the change from baseline analysis.

### Insulin resistance and inflammation markers

The pecan group had a greater reduction from baseline in the LP-IR (–4.42 points; 95% CI: –8.14, –0.69) than the usual diet group (Table 3). Similar results were observed for the LP-IR after 12 wks of pecan consumption compared with the usual diet group (Supplemental Table 2). No between-group differences in the change from baseline or at 12 wks were observed for GlycA, the DRI, or the branched-chain amino acids used to calculate the DRI.

**TABLE 3:** Model-based estimates of the between-group differences in the change from baseline in early markers of insulin resistance in adults at risk of cardiometabolic diseases^1^.

	Pecan		Usual diet		Effect^2^	*P* value^3^
	*n*	Change (95% CI)	*n*	Change (95% CI)		
**Diabetes risk**						
**LP-IR**	62	–1.46 (–4.17, 1.24)	68	2.95 (0.40, 5.50)	–4.42 (–8.14, –0.69)	0.02
**DRI**	62	–0.30 (–3.07, 2.46)	68	2.30 (–0.30, 4.91)	–2.61 (–6.40, 1.19)	0.18
**Branched-chain amino acids(μmol/L)**						
**Valine**	62	2.43 (–5.64, 10.5)	68	3.25 (–4.33, 10.8)	–0.82 (–11.9, 10.2)	0.88
**Leucine**	62	0.21 (–6.00, 6.42)	68	0.40 (–5.43, 6.23)	–0.19 (–8.71, 8.32)	0.96
**Inflammation(μmol/L)**						
**GlycA**	62	–1.36 (–11.8, 9.06)	68	2.46 (–7.35, 12.3)	–3.82 (–18.1, 10.5)	0.60

Abbreviations: CI, confidence interval; DRI, Diabetes Risk Index; LP-IR, Lipoprotein Insulin Resistance Index.

1 Statistical analyses were performed with SAS version 9.4 (SAS Institute). PROC GLM was used to determine between-group differences with adjustment for age, sex, baseline BMI, and baseline value. Data presented as least squares mean difference (95% CI).

2 Between group mean difference in the change from baseline (95% CI).

3 Main effect of randomization.

## Discussion

Replacing usual snacks with 57 g/d of pecans for ∼12 wks reduced the concentration of apoB, total LDL particles, and total, large, and small TRL-P and increased the concentration of large HDL particles and H5 compared with continuing usual intake in adults with increased cardiometabolic risk. Pecan consumption improved the LP-IR compared with the usual diet group, but had no effect on the DRI or GlycA. Collectively, pecan consumption for 3 mo resulted in a less atherogenic lipoprotein particle profile and reduced an early indicator of insulin resistance compared with continuing usual intake.

In this study, intake of pecans reduced the concentration of atherogenic apoB-containing lipoprotein particles. These findings align with the improvements in surrogate markers for apoB (i.e., LDL cholesterol and non-HDL cholesterol) previously observed in this study [20]. The apoB reduction observed in our trial (–4.4 mg/dL) is also consistent with previous research. A meta-analysis of trials including adults with various health statuses (*n* = 8060) showed that a median daily dose of ∼46 g/d of nuts lowered apoB by 4 mg/dL (–6, –2) compared with nut devoid diets after ≥3 wks [25]. All apoB-containing lipoprotein particles are atherogenic because apoB-containing lipoprotein particles can cross the arterial endothelial cell layer and become trapped within the extracellular matrix of the intima in the arterial wall initiating atherosclerotic plaque development [26,27]. When the concentration of apoB-containing particles is low, the likelihood of retention in the intima is also low, but as particles increase, retention increases in a dose-dependent manner [28,29]. In this trial, the improvement in apoB (–4.38 mg/dL) with pecan consumption is expected to be associated with a ∼2.5% reduction in the risk of cardiovascular mortality based on prior evidence [30].

In the fasting state, ∼90% of circulating apoB-containing lipoproteins are LDL particles [28]. In our trial, total LDL particles (–75 nmol/L; 95% CI: –144, –6.93) were reduced in the pecan group compared with the usual diet group; no between-group differences in the concentration of small, medium, or large LDL particles were observed. Consistent evidence shows that all LDL particles cause atherosclerotic CVD, and LDL particles concentration is more strongly associated with atherosclerotic CVD risk than LDL cholesterol [28]. Some evidence suggests small LDL particles are stronger predictors of CVD risk than larger LDL particles, although it is difficult to disentangle the independent effects of 1 class of apoB lipoproteins because of the significant interrelationships between atherogenic apoB-containing lipoproteins [28]. Accumulating evidence suggests that nut intake reduces the concentration of total and small LDL particles [31]. To date, only 2 small randomized controlled trials have investigated the effect of pecan intake on lipoprotein subfractions and the results are inconsistent. One trial showed that intake of 68 g/d of pecans reduced the concentration of total LDL particles (–87 nmol/L) and medium LDL particles (–50 nmol/L) in older adults (*n* = 21–23/group) compared with a diet devoid of nuts [1]. In contrast, in another trial including adults with hypercholesterolemia or a BMI >28 kg/m^2^ (*n* = 16–18/group), intake of 68 g/d of pecans did not affect LDL particle concentration compared with a diet devoid of nuts [4]. These inconsistent results may be related to sample size and statistical power to detect effects for these outcomes.

In this trial, pecan intake increased the concentration of large HDL particles compared with the usual diet group. Epidemiologic evidence supports an inverse association between large HDL particles and CVD risk [[32], [33], [34]]. This is likely explained by the greater cholesterol efflux capacity, a primary component of reverse cholesterol transport, of larger compared with small HDL particles [34]. Recent advances in HDL particle quantification have enabled the assessment of HDL particle subspecies H1 (smallest) through H7 (largest) [22,35]. Limited research has evaluated the association between HDL particle subspecies (H1–H7) and cardiometabolic disease risk. Available evidence from prospective cohort studies including relatively healthy adults suggest that H4 is inversely associated with high-risk coronary atherosclerotic plaque [36], incident myocardial infarction, and ischemic stroke [35], and incident type 2 diabetes [23]. Evidence for other subspecies is inconsistent. In our trial, H4 (medium HDL particle) concentration was lower at 12 wks in the pecan group compared with the usual diet group. We also observed increased H5 (large HDL particle) concentration with pecan intake compared with the usual diet group, but the implications of these findings remain unclear. Given the limited statistical power for these outcomes, findings should be interpreted with caution.

Our results showed pecan intake reduced insulin resistance assessed by the LP-IR. The observed 4.2-point reduction in the LP-IR with pecan intake would be expected to reduce the risk of type 2 diabetes by ∼7% based on available data [15]. The LP-IR comprises 6 lipoprotein parameters (i.e., the size of LDL particles, HDL particles, TRL-P, and the concentration of large TRL-P, small LDL particles, and large HDL particles) altered in insulin-resistant states; we observed improvements for 2 of the 6 components in the pecan groups compared with the usual diet group. The LP-IR is strongly associated with hepatic insulin resistance assessed by HOMA-IR and glucose disposal assessed via hyperinsulinemic–euglycemic clamp, an indicator of peripheral insulin resistance [10,11,37]. We did not observe between-group differences in HOMA-IR at 12 wks (data not reported). Dyslipoproteinemia precedes glycemic changes because of alterations in hepatic lipid metabolism [38,39]. The LP-IR change observed in our trial suggests that pecan intake affected early insulin resistance, without impacting glycemic outcomes including hemoglobin A1c, fasting insulin, or glucose [20].

We did not see the effects of pecan consumption on the DRI, valine or leucine, or GlycA. The GlycA finding aligns with the lack of an intervention effect on C-reactive protein, which was previously reported [20]. Elevated concentrations of valine and leucine are associated with insulin resistance and type 2 diabetes [[40], [41], [42]], but the mechanism and causality are unclear [43,44]. Elevated branched-chain amino acids may impair insulin signaling pathways or, alternatively, could result from dysfunctional catabolism caused by impaired metabolism by insulin-dependent tissue-specific (e.g., adipose and liver) enzymes [44]. The discrepancy between the LP-IR and DRI may be because our cohort had normal baseline concentrations of valine and leucine. Similarly, in our study, participants had GlycA values <400 μmol/L, indicative of low inflammation [45], providing limited room for improvement. Collectively, GlycA, valine, and leucine concentrations were low despite lipoprotein abnormalities characteristic of insulin resistance, which likely explains why only the LP-IR was improved.

Possible contributors to the changes observed in concentrations of LDL particles, TRL-P, apoB, and large HDL particles with pecan consumption compared with usual intake may include PUFA, fiber, phytosterols, and polyphenols including flavan-3-ols and anthocyanins. Pecans contain 12.2 g of PUFA per 57 g [46]. The PUFA content of pecans may have increased LDL particle receptors to improve clearance of LDL particles [47]. It is possible that the reduction in LDL particles and apoB reflects increased receptor-mediated LDL particle clearance. Dietary fiber and phytosterols reduce intestinal cholesterol absorption, which results in decreases in circulating LDL particles [48]. Higher intake of flavan-3-ols and anthocyanins has been associated with lower insulin resistance [[49], [50], [51]] and may have contributed to the improvement in the LP-IR. The combined effect of the pecan components may explain the lipoprotein-related improvements observed.

A strength of this analysis is that most participants had features of or met criteria for MetS. This is a population for which lipoprotein particles provide additional disease risk information. The limitations of this trial include that although all participants had ≥1 feature of MetS at screening, 6 participants did not have any MetS features at baseline, thus compromising the goal of recruiting adults with features of MetS. This was a secondary analysis; therefore, the trial was not powered for these outcomes. The number of analyses conducted increases the risk of type 1 statistical errors.

In conclusion, our results show that replacing usual snacks with 57 g/d of pecans for 12 wks improves the concentration of apoB, total LDL particles, total, large, and small TRL-P, H5, and large HDL particles compared with continuing usual intake in adults at risk for cardiometabolic diseases. Replacement of usual snacks with pecans may reduce insulin resistance, and the risk of atherosclerotic CVD and type 2 diabetes in adults at risk for cardiometabolic diseases.

## Author contributions

The authors’ responsibilities were as follows – KSP, PMK-E: designed the research and critically reviewed the manuscript; TLH: conducted research and drafted the manuscript; TLH, KSP: analyzed and interpreted the data; KSP: had primary responsibility for final content; and all authors: read and approved the final manuscript.

## Data availability

Data described in the manuscript and analytic code will be made available on request.

## Funding

This study was funded by the American Pecan Council. The American Pecan Council had no role in study design, data collection, analysis or interpretation of the data. The project described was supported by the National Center for Advancing Translational Sciences, National Institutes of Health, through grant UL1 TR002014. The content is solely the responsibility of the authors and does not necessarily represent the official views of the NIH.

## Conflicts of interest

KSP received a grant to conduct this research from the American Pecan Council and financial support was provided by the National Center for Advancing Translational Sciences, National Institutes of Health. The authors report no conflict of interest.
