# Supplementary material for: Pecan Intake Improves Lipoprotein Particle Concentrations Compared with Usual Intake in Adults at Increased Risk of Cardiometabolic Diseases: A Randomized Controlled Trial
Source: J Nutr. 2025 Mar 18;155(5):1459–65. doi: 10.1016/j.tjnut.2025.03.014 (PMC12121402; doi:10.1016/j.tjnut.2025.03.014)
Supplement: Multimedia component 1 [file mmc1.docx]

# Supplemental Material

## Supplementary Table 1. Model-based estimates for between-group mean differences for apolipoproteins and lipoproteins in adults at increased risk for cardiometabolic disease^1^

|  | Pecan | | Usual Diet | |  |  |
| --- | --- | --- | --- | --- | --- | --- |
|  | **Baseline**  **(n=69)** | **Endpoint**  **(n=62)** | **Baseline**  **(n=69)** | **Endpoint**  **(n=68)** | **P value^2^** |  |
| Apolipoprotein, mg/dL |  |  |  |  |  | |
| ApoB | 97.9 ± 0.9^a,b^ | 94.6 ± 0.9^a^ | 97.9 ± 0.9^a,b^ | 99.0 ± 0.9^b^ | 0.02 | |
| ApoA1 | 131 ± 1.0 | 135 ± 1.1 | 131 ± 1.0 | 134 ± 1.0 | 0.91 | |
| LDL-P, nmol/L |  |  |  |  |  |  |
| Total^3^ | 1298 (1267, 1330) | 1248 (1216, 1280) | 1300 (1269, 1331) | 1304 (1273, 1336) | 0.08 |  |
| Large^3^ | 262 (234, 292) | 281 (249, 317) | 267 (239, 298) | 252 (225, 281) | 0.26 |  |
| Medium | 345 ± 19.3 | 297 ± 20.2 | 333 ± 19.0 | 337 ± 19.1 | 0.17 |  |
| Small | 658 ± 23.3 | 684 ± 24.5 | 666 ± 23.1 | 677 ± 23.2 | 0.75 |  |
| Triglyceride-Rich Lipoprotein Particle, nmol/L |  |  |  |  |  |  |
| Total | 136 ± 3.3^a,b^ | 125 ± 3.5^a^ | 137 ± 3.3^a,b^ | 145 ± 3.3^b^ | 0.007 |  |
| Very Large^3^ | 0.1 (0.1, 0.2) | 0.2 (0.1, 0.2) | 0.1 (0.1, 0.2) | 0.1 (0.1, 0.2) | 0.18 |  |
| Large^3^ | 1.8 (1.5, 2.3) | 2.0 (1.6, 2.5) | 2.0 (1.6, 2.4) | 2.6 (2.1, 3.2) | 0.38 |  |
| Medium | 16.4 ± 1.0 | 17.5 ± 1.1 | 16.6 ± 1.0 | 20.7 ± 1.0 | 0.14 |  |
| Small | 60.5 ± 2.8 | 52.2 ± 2.9 | 62.1 ± 2.7 | 62.0 ± 2.8 | 0.14 |  |
| Very small^3^ | 44.4 (36.8, 53.5) | 35.1 (28.6, 43.1) | 39.6 (33.3, 47.0) | 39.8 (33.1, 47.7) | 0.20 |  |
| HDL-P, μmol/L |  |  |  |  |  |  |
| Total | 20.7 ± 0.16 | 21.0 ± 0.17 | 20.6 ± 0.16 | 21.0 ± 0.16 | 0.94 |  |
| Large^3^ | 2.0 (1.8, 2.1) | 2.2 (2.0, 2.3) | 2.0 (1.8, 2.1) | 2.0 (1.8, 2.1) | 0.18 |  |
| Medium | 4.1 ± 0.1 | 4.1 ± 0.1 | 4.2 ± 0.1 | 4.4 ± 0.1 | 0.56 |  |
| Small | 14.1 ± 0.2 | 14.2 ± 0.2 | 14.1 ± 0.2 | 14.3 ± 0.2 | 0.70 |  |
| HDL-P subspecies, µmol/L |  |  |  |  |  |  |
| H1 | 3.6 ± 0.1 | 3.8 ± 0.1 | 3.6 ± 0.1 | 3.3 ± 0.1 | 0.08 |  |
| H2 | 10.5 ± 0.2 | 10.4 ± 0.2 | 10.5 ± 0.2 | 10.9 ± 0.2 | 0.12 |  |
| H3 | 1.8 ± 0.1 | 1.9 ± 0.1 | 1.9 ± 0.1 | 1.8 ± 0.1 | 0.40 |  |
| H4 | 2.3 ± 0.1^a,b^ | 2.2 ± 0.1^a^ | 2.3 ± 0.1^a,b^ | 2.6 ± 0.1^b^ | 0.03 |  |
| H5 | 1.1 ± 0.1^a^ | 1.4 ± 0.1^b^ | 1.1 ± 0.1^a^ | 1.0 ± 0.1^a^ | 0.008 |  |
| H6 | 0.8 ± 0.0 | 0.8 ± 0.0 | 0.7 ± 0.0 | 0.8 ± 0.0 | 0.90 |  |
| H7 | 0.5 ± 0.0 | 0.5 ± 0.0 | 0.5 ± 0.0 | 0.5 ± 0.0 | 0.97 |  |
| Average Particle Size, nm |  |  |  |  |  |  |
| LDL^3^ | 21.1 (21.0, 21.1) | 21.0 (20.9, 21.0) | 21.1 (21.0, 21.1) | 21.0 (21.0, 21.1) | 0.25 |  |
| Triglyceride-rich lipoprotein | 43.1 ± 0.6 | 44.1 ± 0.6 | 43.3 ± 0.6 | 44.6 ± 0.6 | 0.74 |  |
| HDL | 9.1 ± 0.0 | 9.2 ± 0.0 | 9.1 ± 0.0 | 9.1 ± 0.0 | 0.50 |  |

^1^Statistical analyses were performed with SAS version 9.4 (SAS Institute). The PROC MIXED procedure was used to determine the main effect of the randomization by visit interaction. When a significant main effect of randomization by visit was detected, post hoc testing was conducted. Results of post hoc testing are denoted with superscript letters. Values within a row without a common letter differ, Tukey adjusted p < 0.05. Data are presented as least-squares means ± SE unless otherwise stated. ApoB, apolipoprotein B; ApoA1, apolipoprotein A1; LDL, low density lipoprotein; HDL, high density lipoprotein. ^2^Main effect of the randomization by visit interaction. ^3^Data were log transformed and are presented as geometric mean (95% CI).

## Supplementary Table 2. Model-based estimates of the between-group mean differences in early markers of insulin resistance in adults at increased risk for cardiometabolic diseases^1^

|  | Pecan | | | | Usual Diet | | | |  | |
| --- | --- | --- | --- | --- | --- | --- | --- | --- | --- | --- |
| μmol/L | n | Baseline | n | Endpoint | n | Baseline | n | Endpoint | P value^2^ | |
| Diabetes Risk |  |  |  |  |  |  |  |  |  |  |
| LP-IR | 69 | 44.2 ± 0.9^a,b^ | 62 | 43.0 ± 1.0^a^ | 69 | 44.5 ± 0.9^a,b^ | 68 | 47.0 ± 0.9^b^ | 0.04 |  |
| DRI | 69 | 35.6 ± 0.9 | 62 | 35.5 ± 1.0 | 69 | 35.8 ± 0.9 | 68 | 37.8 ± 0.9 | 0.27 |  |
| Branched Chain Amino Acids |  |  |  |  |  |  |  |  |  |  |
| Valine | 69 | 214 ± 2.7 | 62 | 216 ± 2.9 | 69 | 214 ± 2.7 | 68 | 217 ± 2.7 | 0.85 |  |
| Leucine | 69 | 111 ± 2.1 | 62 | 111 ± 2.2 | 69 | 111 ± 2.1 | 68 | 111 ± 2.1 | 0.94 |  |
| Inflammation |  |  |  |  |  |  |  |  |  | |
| GlycA | 69 | 381 ± 3.7 | 62 | 382 ± 3.8 | 69 | 382 ± 3.6 | 68 | 385 ± 3.6 | 0.82 | |

^1^Statistical analyses were performed with SAS version 9.4 (SAS Institute). The PROC MIXED procedure was used to determine the main effect of the randomization by visit interaction. When a significant main effect of randomization by visit was detected, post hoc testing was conducted. Results of post hoc testing are denoted with superscript letters. Values within a row without a common letter differ, Tukey adjusted p < 0.05. Data are presented as least-squares means ± SE. LP-IR, Lipoprotein Insulin Resistance Index; DRI, diabetes risk index. ^2^Main effect of the randomization by visit interaction.
